# Whooping Cough in New York

**Published:** 1914-05

**Authors:** 


WHOOPING COUGH IN NEW YORK.—A plan has been developed for the supervision and the treatment of cases of whooping cough by the hospitals and dispensaries. A survey has recently been completed of the institutions, hospitals, and dispensaries of New York with a view to determining what institutions treat whooping cough at the present time, what institutions desire to maintain whooping cough clinics, and what facilities they possess for dealing with this disease. Altogether, 116 were visited; of these seventy-eight reported that they have no cases of whooping cough applying for treatment; only five have a district physician attending whooping cough patients at their homes.

---

THE VIRGINIA LEGISLATURE has repealed the law requiring a State license and fee from physicians practicing within the State, the chief argument in support of the repeal dwelling upon the important function of the physician as a field agent of the health authorities. This argument was a good one, but it is interesting to note that the favorable result was accomplished by a well organized profession. A majority of the members of the assembly were pledged to the passage of the bill before that body met.

---

BACKWARD, turn backward, O time, in your flight; give me a girl whose skirts aren't too tight. Give us a girl whose charms (generally few) are not exposed by a large peekaboo. Give us a girl (no matter what age), who knows that a street is no vaudeville stage. Give us a girl who is not all in view—dress her in skirts the sun can't shine through.—*Exchange*.

---

"Whatever the weather may be," says he,
"Whatever the weather may be,
It's the song ye sing, an' the smiles ye wear,
That's a-makin' the sun shine everywhere."

*James Whitcomb Riley.*

---

Be we financier, industrial worker, navvy, scavenger, or merely a gentleman, we all suffer to a greater or less degree from the ills which our vocations or avocations engender. Just how to prevent, ameliorate or cure these but partly understood ills, should no longer be a puzzle to the man, employer or physician because a reliable and essentially practical book is soon to appear, under the joint editorship of Dr. George M. Kober of Washington, D. C., and Dr. Wm. C. Hanson of Boston, Mass. Among the contributors are such authorities as Sir Thomas Oliver; Legge (London); Teleky (Vienna); Devoto (Milan); Edsall (Harvard); Alice Hamilton (Chicago); etc., etc.

P. Blakiston's Son & Co., Philadelphia, will publish the volume.

